# Determinants of engagement in muscle-strengthening activities among children and adolescents: insights from a large-scale school-based study

**DOI:** 10.3389/fpubh.2025.1526907

**Published:** 2025-04-08

**Authors:** Hongtao Sheng, Bin Ge, Yang Li, Zehan Xu, Xingyi Yang, Wenjun Wang, Jin Yan

**Affiliations:** ^1^School of Physical Education, Chifeng University, Chifeng, China; ^2^Department of Physical Education and Sports, Nanjing Medical University, Nanjing, China; ^3^College of Sports Science, Shenyang Normal University, Shenyang, China; ^4^Faculty of Science, University of Sydney, Sydney, NSW, Australia; ^5^Centre for Mental Health, Shenzhen University, Shenzhen, China; ^6^School of Physical Education and Sports Science, Soochow University, Suzhou, China

**Keywords:** muscle-strengthening activity, correlation, sociodemographic, self-perception, China

## Abstract

**Objectives:**

This study examined the sociodemographic factors associated with muscle-strengthening exercise (MSE) participation in a sample of Chinese school-aged children.

**Methods:**

A cross-sectional survey was conducted in March 2021, comprising 67,281 students from public schools in Shenzhen through a multistage sampling method. The survey collected data on MSE participation, sociodemographics, family and educational information, and other aspects. A three-level mixed multilevel effect model was performed to analyze the associations between the selected sociodemographic characteristics and MSE participation. Results were presented using odd ratios (OR) with 95% confidence intervals (CI).

**Results:**

38.6% of participants met the MSE guidelines. In models of adherence to MSE guidelines and MSE days, boys were more likely to participate in MSE (OR = 1.31, 95%CI: 1.27–1.36, *p* < 0.001) than girls. Compared to primary school students, junior middle school students (OR = 1.60, 95%CI: 1.47–1.74, *p* < 0.001) were more likely to participate in MSE, whereas high school students (OR = 0.61, 95%CI: 0.51–0.73, *p* < 0.001) participated less frequently. Socioeconomic status (both OR = 1.07, 95%CI: 1.01–1.13, *p* < 0.001) was positively associated with MSE participation. Participants who perceived their weight as “about the right weight” or “slightly overweight” were most likely to participate in MSE. Weight satisfaction was negatively associated with guideline adherence, with those “very dissatisfied” with their weight being more likely to adhere. Positive associations between sports club participation and sports equipment satisfaction with MSE participation were also found. Other factors, such as ethnicity and parental education level, showed no significant association.

**Conclusion:**

MSE participation is correlated by sociodemographic, behavioral, and self-perception factors, particularly sex, age, socioeconomic status, sports club involvement, and weight perception. Interventions to enhance MSE participation should target the important identified factors. Future studies should consider using longitudinal designs to strengthen understanding of MSE, further aiding in developing relevant and effective interventions.

## 1 Introduction

Muscle-strengthening exercise (MSE) is crucial for children and adolescents’ healthy development and growth (1, 2). Engaging in MSE improves cardiorespiratory fitness and musculoskeletal strength, positively impacts cognitive development, reduces the risk of sports-related injuries and obesity, and decreases the development of mental health issues (3–6). Given these benefits, the World Health Organization (WHO) advises that children and adolescents aged 5–17 should participate in specifically designed activities that strengthen muscles and bones at least three times per week (7–10).

Despite the evidence-based recommendation on MSE participation, the prevalence rates of the MSE guidelines remain low in children and adolescents worldwide. Data from a US population-level survey reveals that only about 50% of adolescents meet the MSE guidelines (11, 12). Notably, adherence rates are even lower in socioeconomically disadvantaged regions, highlighting significant disparities in MSE participation in various demographic groups (3–5, 13, 14). These disparities present a significant public health concern, emphasizing the urgent need for effective strategies to increase MSE participation among children and adolescents.

Extant research predominantly focuses on adult populations (6, 14), while research attention is relatively less focused on young populations. This results in a significant research gap in understanding MSE participation in children and adolescents. This gap highlights the need for targeted studies on younger populations developing habits impacting their lifelong health and physical activity patterns. Therefore, gaining deeper insights into the specific factors that encourage or impede MSE participation in children and adolescents in various cultural and socioeconomic contexts is essential.

Several studies have explored factors associated with MSE participation in children and adolescents according to the Social Ecological Model—a well-developed and practical theoretical framework for understanding health behaviors (15). These factors can be categorized into intrapersonal (e.g., sociodemographic, self-perception characteristics), interpersonal, community, organizational, and public policy levels. At the sociodemographic level, some studies have indicated that sex, age (grade), ethnicity, and family socioeconomic status are significant factors related to MSE participation (4, 5, 16, 17). For example, Chen et al. (5) reported that boys were significantly more involved in MSE participation than girls. Xin et al. (4) found that high school students participated less in MSE than primary and middle school students. Wilson et al. (16) indicated that Asian adolescents have lower odds of meeting MSE guidelines than their European peers, while Pasifika males are more likely to meet these guidelines than European males. They also highlighted that children and adolescents from higher socioeconomic status areas had higher odds of meeting MSE guidelines than those from low socioeconomic status areas (16).

In addition to sociodemographic factors, evidence has demonstrated the association between self-perception-related characteristics and MSE participation, such as perceived weight, satisfaction with weight, and body image (18, 19). These characteristics can change individual behaviors through complex social and cognitive interactions. Martini et al. (20) found that higher satisfaction with weight was linked to increased MSE participation in children and adolescents. Shi et al. (21) reported that adolescents who perceived themselves as overweight participated significantly less in MSE. Effa et al. (22) also found that a positive body image was associated with higher MSE participation.

Family-related factors also play a crucial role in influencing MSE participation in children and adolescents. Research indicates that the presence of siblings, parental education levels, and living arrangements are significantly associated with MSE participation. These family-related attributes provide various supports, resources, and encouragements that can promote regular MSE participation and maintenance. However, mixed results remained in the literature. Xin et al. (4) reported that adolescents with siblings participated more in MSE. Gu et al. (3) found that parental education was not associated with MSE participation. Wang et al. (23) highlighted that adolescents living with both parents had higher prevalence rates of MSE participation compared to those living with a single parent.

Furthermore, evidence has shown that some specific behaviors are interrelated and codependent among various healthy behaviors. From this perspective, some factors of exercise participation are associated with MSE participation. For example, Parker et al. (24) reported that adolescents satisfied with their exercise equipment were significantly more likely to participate in MSE. Toivo et al. (25) found that adolescents involved in sports clubs had higher MSE participation rates compared to those not involved in such clubs.

Although previous studies [e.g., (3, 4)] have examined the participation rate and correlates of MSE among Chinese children and adolescents, specific research gaps remain unaddressed. Most existing studies on MSE participation have been conducted in Western countries, such as Australia and the United States, highlighting the need for more research focused on Chinese children and adolescents. While previous China-based studies primarily explored sociodemographic factors, they did not comprehensively examine multidimensional influences on MSE participation. For instance, Xin et al. mainly focused on the prevalence of meeting MSE recommendations and its associations with demographic, behavioral, psychological, and sociocultural factors but did not consider the impact of self-perception factors (e.g., weight perception and satisfaction), sports club participation, or satisfaction with sports equipment on MSE engagement. Additionally, Gu et al. conducted their study on a relatively small sample (*n* = 3,733) from Hubei Province, limiting the generalizability of their findings to a broader national context. In contrast, this study is based on a more extensive and diverse dataset, offering a more comprehensive analysis of the multidimensional factors influencing MSE participation. Therefore, this study aimed to investigate the various factors related to MSE participation in children and adolescents in a large sample of school students.

## 2 Materials and methods

### 2.1 Study design and participants

In collaboration with the Municipal Education Commission, a large-scale survey was conducted in primary, middle and high schools in Shenzhen, China, in March 2021. The sample included students from local public primary and secondary schools from all districts of Shenzhen. Previous PA-related studies in Chinese children and adolescents suggested that children 10 years or older had sufficient reading comprehension and the ability to complete the self-report questionnaire (26, 27). Also, the Municipal Education Commission recommended surveying school-going students of grades five and above. Consequently, students in grades five and above were considered eligible study participants. Accordingly, the study included primary, junior, middle, and high school students.

Students in grades 9 and 12 were excluded from the survey due to their preparations for crucial entrance exams. We selected students aged 10 years and older for their cognitive ability to self-report via questionnaires. All students and their guardians were informed about the survey’s purpose, and participation was voluntary and anonymous. The survey was conducted online in computer rooms, taking approximately 20 min during a school day. Only students who provided electronic informed consent were included in the data collection phase. This study received ethical approval from the Medical Ethics Committee of Shenzhen University (No. 2020005) and the endorsement of teachers and principals at the participating schools.

Out of 79,664 targeted children and adolescents, 78,428 submitted questionnaires, yielding a response rate of 98.4%. After removing invalid data (e.g., responses failing quality checks), 73,323 samples from 135 schools constituted our preliminary database. For this study, we analyzed data from 67,281 students based on the availability of necessary variables.

For MSE data collection, we asked students: “In the last 7 days, how much time did you spend on exercises to tone or strengthen muscles, such as lifting weights, doing sit-ups, or push-ups?” Response options ranged from 0 to 7 (every day) (28). This item, used in health behavior surveillance in other countries (29), demonstrated acceptable reliability for children and adolescents with a Kappa coefficient greater than 0.55 (30). Consistent with World Health Organization guidelines, children and adolescents who participated in MSE for at least 3 days a week were considered to meet the recommendations (26, 31).

Additionally, the following information was gathered: sex (boy/girl), age (10–17 years old), grade (primary school/junior middle school/high school), ethnicity (Han/minority), number of siblings (only child/not only child), living with a parent (yes/no), a parental education level (junior middle school or below/high school or equivalent/bachelor or equivalent/master or above/unclear), self-perceived weight (very underweight/slightly underweight/about the right weight/slightly overweight/very overweight) (32), satisfaction with weight (very dissatisfied/dissatisfied/neither satisfied nor dissatisfied/satisfied/very satisfied), satisfaction with sports equipment (very satisfied/satisfied/dissatisfied/very unhappy/not sure), sports club participation (never/1–3 times per month/1–2 times per week/3 or more times per week), and family socioeconomic status (SES) (21). SES was measured using an adapted version of the MacArthur Scale of Subjective Social Status (a 10-rung ladder with higher scores indicating better SES) (33).

### 2.2 Statistical analysis

**Figure 1:**
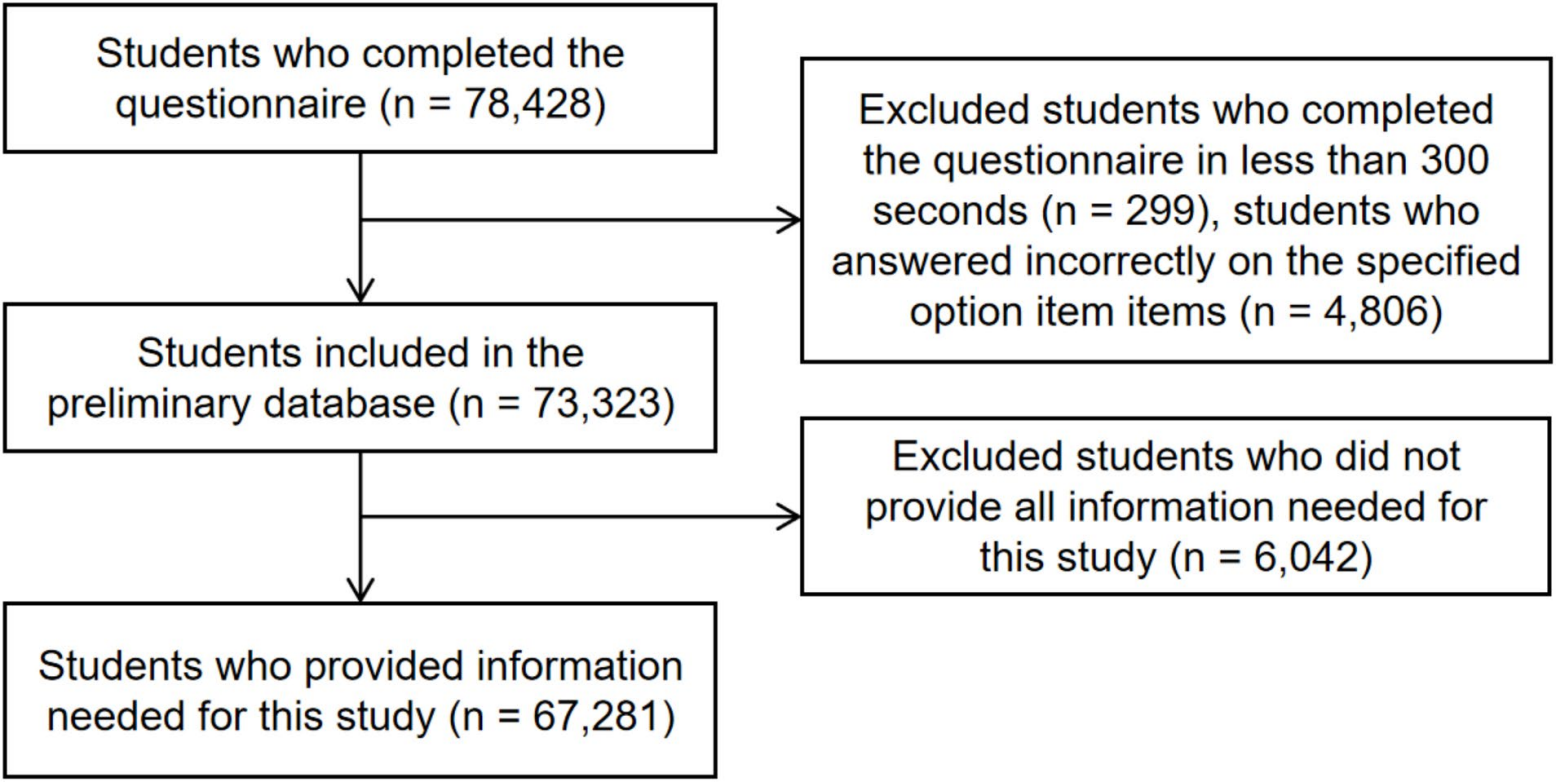
Detailed process used for cleaning invalid and missing data.

A total of 67,281 participants were included in the final formal analysis after excluding samples with invalid or incomplete data (*n* = 11,147). The process can be seen in Figure 1. All statistical analyses were conducted using STATA BE 18.0 (College Station, Texas, United States). We used descriptive statistics to summarize sample characteristics and employed a three-level mixed multilevel effect model to analyze the data. This reflects our multistage sampling strategy (level 3: district; level 2: school; level 1: individual). We analyzed the factors associated with MSE in children and adolescents. The analysis included logistic regression models to examine the associations between sociodemographic variables (such as sex, age, socioeconomic status, parental education, and participation in sports clubs) and MSE guidelines or days. The models were adjusted for potential confounders, including the district and school levels. Results are presented as odds ratios (OR) with 95% confidence intervals (CI), and statistical significance was assessed at a *p*-value of less than 0.05.

## 3 Results

Table 1 presents the sample characteristics of the study participants, including 34,909 boys (51.9%) and 32,372 girls (48.1%) aged 10–17 years. In terms of weight perception, 45.2% of participants believed they were “about the right weight,” 31.8% considered themselves “slightly overweight,” and 14.9% viewed themselves as “slightly underweight.” Participant satisfaction with their weight varied, with 30.7% being dissatisfied, 31.4% remaining neutral, and 13.3% feeling very satisfied. Most study participants (74.2%) had siblings, and most (93.4%) lived with both parents. Regarding parental education, 27.0% of fathers and 27.8% of mothers achieved “high school or equivalent” as their highest educational qualification. Satisfaction with sports equipment was relatively high, with 45.8% satisfied and 31.8% very satisfied. 47.1% did not participate in sports clubs, while 13.0% attended thrice weekly. The educational levels of participants spanned primary (41.5%), junior middle (40.3%), and high schools (18.1%). The Han ethnicity predominated at 96.6. The mean of subjective family socioeconomic status was 4.95 on a scale from 0 to 10. The prevalence rate of meeting the MSE guidelines was 38.6%; also, the prevalence rate of days of MSE ranged from being the most of 24.9 (0 days) to the lowest of 1.6% (6 days). More details on sample characteristics can be found in Table 1.

**Table 1:** Descriptive characteristics of participants.

Categorical variables		*n*	%
Sex			
	Boy	34,909	51.9%
	Girl	32,372	48.1%
Age			
	10 years old	2,430	3.6%
	11 years old	10,933	16.2%
	12 years old	16,244	24.1%
	13 years old	15,331	22.8%
	14 years old	8,871	13.2%
	15 years old	4,076	6.1%
	16 years old	6,379	9.5%
	17 years old	3,017	4.5%
Grade			
	Primary school	27,954	41.5%
	Junior middle school	27,124	40.3%
	High school	12,203	18.1%
Ethnicity			
	Han	65,027	96.6%
	Minority	2,254	3.4%
Siblings			
	Only child	17,354	25.8%
	Non-only child	49,927	74.2%
Were you living with a parent?			
	Yes	62,836	93.4%
	No	4,445	6.6%
Paternal education			
	Junior middle school or below	14,619	21.7%
	High school or equivalent	18,159	27.0%
	Bachelor or equivalent	26,030	38.7%
	Master or above	2,796	4.2%
	Unclear	5,677	8.4%
Maternal education			
	Junior middle school or below	17,617	26.2%
	High school or equivalent	18,706	27.8%
	Bachelor or equivalent	23,922	35.6%
	Master or above	1,635	2.4%
	Unclear	5,401	8.0%
How did you describe your weight?			
	Very underweight	1,912	2.8%
	Slightly underweight	10,010	14.9%
	About the right weight	30,444	45.2%
	Slightly overweight	21,401	31.8%
	Very overweight	3,514	5.2%
How satisfied were you with your weight?			
	Very dissatisfied	7,645	11.4%
	Dissatisfied	20,678	30.7%
	Neither satisfied nor dissatisfied	21,107	31.4%
	Satisfied	8,874	13.2%
	Very satisfied	8,977	13.3%
How satisfied were you with the equipment for sport activity?			
	Very satisfied	21,405	31.8%
	Satisfied	30,829	45.8%
	Dissatisfied	9,796	14.6%
	Very dissatisfied	3,153	4.7%
	Not sure	2,098	3.1%
Sport club			
	Never	31,721	47.1%
	1–3 times per month	15,050	22.4%
	1–2 times per week	11,789	17.5%
	3 or more times per week	8,721	13.0%
Muscle-strengthening exercise days			
	0 days	16,780	24.9%
	1 day	12,164	18.1%
	2 days	12,393	18.4%
	3 days	10,725	15.9%
	4 days	4,521	6.7%
	5 days	5,335	7.9%
	6 days	1,070	1.6%
	7 days	4,293	6.4%
Muscle-strengthening exercise guideline (3 or more days)			
	Not meet	41,337	61.4%
	Meet	25,944	38.6%
Continuous variables		Mean	SD
Subjective family socioeconomic status		4.95	0.01

SD, standard deviation. Subjective family socioeconomic status was scaled from 0 to 10.

Table 2 shows results on the association between sample characteristics and adherence to MSE guidelines. Boys were significantly more likely (OR = 1.31, 95%CI: 1.27–1.36, *p* < 0.001) to meet these guidelines than girls. Self-weight perception was significantly associated with MSE guidelines. Compared with those who self-described as “very overweight,” those who self-reported “about the right weight” (OR = 1.29, 95%CI: 1.18–1.42, *p* < 0.001) or “slightly overweight” (OR = 1.12, 95%CI: 1.02–1.23, *p* = 0.013) was more likely to adhere the guidelines. There was also a significant relationship between weight satisfaction and guideline adherence. Those who were “dissatisfied” (OR = 0.92, 95%CI: 0.86–0.98, *p* = 0.011), “neither satisfied nor dissatisfied” (OR = 0.92, 95%CI: 0.86–0.99, *p* = 0.020), and “satisfied” (OR = 0.88, 95%CI: 0.82–0.96, *p* = 0.002) with their weight were less likely to meet the guidelines compared to those who were “very dissatisfied.”

**Table 2:** Associations between meeting muscle-strengthening exercise guidelines with characteristics.

Meeting muscle-strengthening exercise guidelines	OR	95% CI		*p*
Sex				
Girl	Reference			
Boy	1.31	1.27	1.36	<0.001
Grade				
Primary school	Reference			
Junior middle school	1.83	1.66	2.02	< 0.001
High school	0.53	0.43	0.66	< 0.001
Ethnicity				
Han	Reference			
Minority	0.95	0.86	1.04	0.286
Siblings				
Non-only child	Reference			
Only child	1.06	1.02	1.10	0.006
Were you living with a parent?				
No	Reference			
Yes	1.05	0.97	1.12	0.215
Subjective family socioeconomic status	1.07	1.06	1.08	<0.001
Paternal education				
Junior middle school or below	Reference			
High school or equivalent	1.07	1.01	1.13	0.025
Bachelor or equivalent	0.99	0.93	1.05	0.803
Master or above	1.07	0.96	1.19	0.220
Unclear	0.98	0.89	1.09	0.757
Maternal education				
Junior middle school or below	Reference			
High school or equivalent	1.03	0.98	1.09	0.280
Bachelor or equivalent	1.02	0.96	1.08	0.542
Master or above	1.07	0.94	1.21	0.338
Unclear	1.01	0.91	1.12	0.900
How did you describe your weight?				
Very overweight	Reference			
Slightly overweight	1.12	1.02	1.23	0.013
About the right weight	1.29	1.18	1.42	<0.001
Slightly underweight	1.21	1.09	1.33	<0.001
Very underweight	1.16	1.02	1.32	0.029
How satisfied were you with your weight?				
Very dissatisfied	Reference			
Dissatisfied	0.92	0.86	0.98	0.011
Neither satisfied nor dissatisfied	0.92	0.86	0.99	0.020
Satisfied	0.88	0.82	0.96	0.002
Very satisfied	1.08	1.00	1.17	0.058
How satisfied were you with the equipment for sport activity?				
Not sure	Reference			
Very satisfied	1.94	1.74	2.16	<0.001
Satisfied	1.50	1.35	1.67	<0.001
Dissatisfied	1.41	1.26	1.58	<0.001
Very dissatisfied	1.43	1.25	1.63	<0.001
Sport club participation				
Never	Reference			
1–3 times per month	1.28	1.23	1.34	<0.001
1–2 times per week	1.39	1.33	1.46	<0.001
3 or more times per week	3.04	2.88	3.21	<0.001

OR, odds ratio; CI, confidence interval. Models controlled for age group.

Being an only child was associated with higher odds of meeting the guidelines (OR = 1.06, 95%CI: 1.02–1.10, *p* = 0.006). Only paternal education at the “high school or equivalent” level (OR = 1.07, 95%CI: 1.01–1.13, *p* < 0.001) was significantly associated with guideline adherence, and other parental education was not associated with guideline adherence. A higher family SES was positively associated with guideline adherence, with an OR of 1.07 with 95%CI between 1.01 to 1.13. Living with parents did not show a significant association with guideline adherence. However, satisfaction with sports equipment and participation in sports clubs were both positively correlated with guideline adherence, particularly among those “very satisfied” (OR = 1.43, 95%CI: 1.25–1.63, *p* < 0.001) with their equipment and those participating in clubs more frequently. Educational levels revealed that junior middle school students (OR = 1.83, 95%CI: 1.66–2.02, *p* < 0.001) were more likely to adhere to the guideline compared to primary school students, whereas high school students (OR = 0.53, 95%CI: 0.43–0.66, *p* < 0.001) showed lower guideline adherence. There were no significant differences in the guideline adherence across ethnicity or whether they lived with their parents.

Table 3 displays the association between MSE participation days and various sample characteristics. Boys had significantly more MSE days (OR = 1.34, 95%CI: 1.31–1.38, *p* < 0.001) than girls. Weight perception was significantly associated with MSE days; participants who considered themselves “about the right weight” (OR = 1.33, 95%CI: 1.23–1.44, *p* < 0.001) or “slightly overweight” (OR = 1.15, 95%CI: 1.07–1.24, *p* < 0.001) participated more frequently in MSE than those who viewed themselves as “very underweight.” Interestingly, those who were “very satisfied” (OR = 1.12, 95%CI: 1.05–1.19, *p* = 0.001) with their weight were less likely to participate in these activities compared to their “very dissatisfied” counterparts. The presence of siblings was associated with slight increases in days of MSE participation (OR = 1.06, 95%CI: 1.03–1.10, *p* < 0.001). Only paternal education at the “high school or equivalent” level (OR = 1.09, 95%CI: 1.04–1.14, *p* < 0.001) and maternal education at the “master or above” level (OR = 1.15, 95%CI: 1.04–1.28, *p* = 0.009) was positively associated with more MSE days. Moreover, a higher family SES was positively associated with days of MSE participation, showed by OR of 1.07 with 95%CI between 1.06 and 1.08 (*p* < 0.001). Living with a parent marginally increased the likelihood of MSE days (OR = 1.12, 95%CI: 1.05–1.18, *p* < 0.001). Satisfaction with sports equipment emerged as a strong predictor; those “very satisfied” (OR = 2.02, 95%CI: 1.86–2.19, *p* < 0.001) with their equipment were much more likely to participate frequently in such activities compared to those who were “not sure.” Participation in sports clubs was a robust indicator of higher engagement, with attendees of clubs “3 or more times per week” showing significantly higher odds (OR = 3.24, 95%CI: 3.10–3.30, *p* < 0.001) of frequent participation compared to non-attendees. Educational levels were associated with MSE participation as well, with junior middle school students (OR = 1.60, 95%CI: 1.47–1.74, *p* < 0.001) participating more than primary school students, whereas high school students (OR = 0.61, 95%CI: 0.51–0.73, *p* < 0.001) participated the lowest. Lastly, ethnicity does not show a significant association with MSE participation.

**Table 3:** Associations between days of muscle-strengthening exercise participation with characteristics.

Muscle-strengthening exercise days	OR	95% CI		*p*
Sex				
Girl	Reference			
Boy	1.34	1.31	1.38	<0.001
Grade				
Primary school	Reference			
Junior middle school	1.60	1.47	1.74	<0.001
High school	0.61	0.51	0.73	<0.001
Ethnicity				
Han	Reference			
Minority	0.98	0.91	1.06	0.633
Siblings				
Non-only child	Reference			
Only child	1.06	1.03	1.10	<0.001
Were you living with a parent?				
No	Reference			
Yes	1.12	1.05	1.18	<0.001
Subjective family socioeconomic status	1.07	1.06	1.08	<0.001
Paternal education				
Junior middle school or below	Reference			
High school or equivalent	1.09	1.04	1.14	<0.001
Bachelor or equivalent	1.03	0.98	1.08	0.242
Master or above	1.04	0.95	1.14	0.364
Unclear	1.02	0.94	1.11	0.638
Maternal education				
Junior middle school or below	Reference			
High school or equivalent	1.03	0.98	1.07	0.216
Bachelor or equivalent	1.02	0.97	1.07	0.417
Master or above	1.15	1.04	1.28	0.009
Unclear	0.95	0.88	1.04	0.287
How did you describe your weight?				
Very overweight	Reference			
Slightly overweight	1.15	1.07	1.24	<0.001
About the right weight	1.33	1.23	1.44	<0.001
Slightly underweight	1.23	1.13	1.33	<0.001
Very underweight	1.21	1.09	1.35	<0.001
How satisfied were you with your weight?				
Very dissatisfied	Reference			
Dissatisfied	0.96	0.91	1.01	0.107
Neither satisfied nor dissatisfied	0.96	0.91	1.01	0.120
Satisfied	0.94	0.88	1.00	0.063
Very satisfied	1.12	1.05	1.19	0.001
How satisfied were you with the equipment for sport activity?				
Not sure	Reference			
Very satisfied	2.02	1.86	2.19	<0.001
Satisfied	1.62	1.50	1.76	<0.001
Dissatisfied	1.47	1.35	1.61	<0.001
Very dissatisfied	1.33	1.20	1.48	<0.001
Sport club participation				
Never	Reference			
1–3 times per month	1.45	1.40	1.50	<0.001
1–2 times per week	1.59	1.54	1.66	<0.001
3 or more times per week	3.24	3.10	3.39	<0.001

OR, odds ratio; CI, confidence interval. Models controlled by age group.

## 4 Discussion

Using a large sample of Chinese children and adolescents, this study found that (1) the prevalence of meeting the MSE recommendation was relatively low; (2) significant correlates of meeting the MSE recommendation and days of MSE participation were sex, age (grade), family SES, siblings, perceived weight, and sports clubs.

Our results indicated that many children and adolescents needed to meet the MSE guidelines. This finding aligns with some previous studies (3, 4). This finding highlights the need for effective public health interventions to promote MSE participation in children and adolescents. Schools should integrate structured MSE programs and provide the necessary resources for enhancement. Public health policies should raise awareness about MSE benefits and ensure accessible facilities. Future research should explore effective interventions to increase MSE participation and examine long-term health outcomes, such as reduced chronic disease risk and improved overall well-being.

Our results indicated that boys were more likely than girls to participate in MSE, consistent with previous studies by Bennie et al. (13), Xin et al. (4), Smith et al. (34), and Gu et al. (3). The sex difference in MSE participation could be attributed to several interconnected factors. Social and cultural norms often dictated different expectations and opportunities for PA between sexes. Boys were typically encouraged to participate in strength-building activities, while girls were often directed toward more sedentary pursuits, such as reading and drawing (35, 36). Additionally, sports programs and facilities in schools and communities tend to cater more to interests traditionally associated with boys, possibly boosting their MSE participation rates (37, 38). Furthermore, girls faced unique barriers, such as limited social support, lower self-efficacy concerning strength activities, and body image concerns (4, 37, 39), which further likely reduced their MSE participation. These insights highlighted the need for targeted interventions to encourage MSE participation among girls.

Our study indicated that MSE participation increased notably as students transitioned from primary to junior middle school but declined significantly by high school, similar to the findings of Xin et al. (4). The shifting academic and sociocultural contexts in China may explain this pattern. In primary school, students generally experience fewer academic pressures, allowing more opportunities for MSE participation. However, their exposure to structured and diverse physical activities (PA) may still be limited, potentially hindering motor skill development and physical confidence necessary for regular participation (40). In junior middle school, students typically gain greater autonomy and physical capability, which, combined with broader access to sports and structured activities, contributes to increased MSE participation (41). However, as students progress to high school, the emphasis on academic achievement intensifies, primarily due to the national college entrance examination system. High school students must complete a substantial volume of educational assignments, with their performance heavily dependent on study time rather than physical activity (42). Moreover, the demanding academic workload limits students’ available time for physical activity and adequate sleep and shifts their priorities away from health-related behaviors, further constraining MSE participation (43). High school students often perceive MSE as a low-priority activity, as it does not directly contribute to their academic performance. This mindset and limited institutional support for non-academic pursuits exacerbate the decline in MSE participation. Given these trends, future studies should adopt longitudinal designs to explore further the relationship between academic demands and MSE participation across different educational stages.

Our study found that sports club participation was positively associated with MSE participation in children and adolescents, which is consistent with the study by Toivo et al. (25). Sports clubs provide structured environments that foster regular MSE participation by offering social support, coaching, and skills development, all crucial for maintaining interest and MSE participation (28, 38). This can also be reflected in the positive association between satisfaction with sports equipment and MSE participation in the current study. They also promoted routine and commitment, essential for establishing lasting exercise habits (44). Conversely, lower rates of MSE participants in non-participants highlighted potential access or interest barriers, underscoring the need for inclusive programs that accommodate various backgrounds and abilities. The social environment of sports clubs, including peer relationships and a sense of team belonging, played a vital role in motivating children, particularly during early adolescence when peer influence peaks (28, 45). This supported policy advocacy aimed at broadening access to sports clubs to boost MSE participation in children and adolescents.

Our study found that higher SES was positively associated with more MSE participation. This observation is consistent with broader public health research identifying socioeconomic factors as determinants of health behaviors, including MSE participation (4, 23, 46). Higher SES likely facilitated greater access to resources such as sports equipment, recreational facilities, sports clubs, and extracurricular opportunities, encouraging MSE participation (4, 24). Families with higher SES often value and support health-promoting behaviors, including MSE, and have better health literacy and education, enhancing their use of health resources (45, 47). Higher SES neighborhoods typically provided safer environments and better infrastructure for MSE participation, such as well-maintained parks and sports fields (48). This relationship highlighted the critical role of socioeconomic factors in shaping access to and engagement in health-promoting activities.

Our results found that parental education was primarily not associated with MSE participation in children and adolescents, contrary to previous research suggesting that educated parents encourage their children to participate in MSE due to a better understanding of PA (4, 13). In the current study, only paternal education at the “High school or equivalent” level showed a slight positive link. In contrast, maternal education showed limited relevance except at the “Master or above” level, slightly related to MSE days. The anticipated association between higher parental education levels and increased MSE participation was not observed in the previous literature (3, 4, 13, 24), possibly due to the sample characteristics, with many parents having lower education levels. Additionally, higher-educated parents might have more demanding jobs, limiting their time to promote MSE participation in children and adolescents (49).

Our study revealed that children and adolescents who perceive themselves as “about the right weight” were more likely to participate in MSE than those who saw themselves as “very underweight” or “very overweight.” Unlike Shi et al. (21), who found that self-perceived as obese individuals were more likely to meet MSE guidelines. Self-perception of a healthy weight may enhance motivation to engage in PA to maintain perceived health (39). Those who viewed themselves as “very underweight” or “very overweight” were less likely to participate, possibly due to negative body images (50), lack of confidence, physical discomfort, or fear of judgment (51). Interestingly, those identifying as “slightly overweight” still actively participated in MSE, recognizing the importance of PA for weight management (52). These findings highlight the need to use self-perception weight and the psychological approaches to MSE interventions.

Our study challenged the assumption that only children engage in less PA due to the lack of siblings (4). Instead, we found that only children were more likely to participate in MSE. This might be attributed to greater parental attention and resources, such as transportation to sports facilities or club memberships, facilitating MSE participation. Additionally, only children may receive more focused encouragement from parents to engage in organized sports for socialization, potentially compensating for the absence of sibling interactions (53). However, this finding contrasts with previous studies suggesting that siblings positively influence PA participation through shared activities, peer modeling, and reinforcement of active lifestyles (54–56). One possible explanation for this discrepancy is that parental investment in children’s extracurricular activities, including MSE, may be more pronounced in single-child households, where resources such as time, financial support, and parental involvement are concentrated on one child rather than divided among multiple siblings.

In contrast, in multi-child households, parental resources may be distributed among various children, potentially reducing the level of individualized support for MSE participation. Furthermore, previous studies may have focused on broader PA behaviors, whereas our research specifically examined MSE participation. The structured nature of MSE might make it more accessible to only children who receive parental encouragement and logistical support, whereas informal, unstructured PA—where siblings may have a stronger influence—could show different trends. The modest association we observed suggests that while being an only child may provide more opportunities and support for MSE participation, it is not the sole determining factor. Other familial and environmental influences, such as parental attitudes toward sports, school PE policies, and access to community sports programs, likely contribute to shaping children’s participation in MSE (57, 58). Given these nuanced findings, future research should explore the mechanisms underlying the relationship between family structure and MSE participation in greater depth, considering parental involvement, socioeconomic background, and cultural expectations. Longitudinal studies examining changes in MSE participation as family dynamics evolve would help clarify the causal pathways and inform targeted interventions to promote PA and MSE participation among all children, regardless of sibling status.

We found that the associations of living with parents and satisfaction with weight with MSE participation varied according to the measures of MSE. For example, the association between living with parents and MSE guideline adherence was significant, whereas the association was not significant when using days of MSE participation as the outcome. Possible reasons could be that different measurements resulted in a sample distribution that affected the significance of the statistical analysis. Living with parents may enhance parental supervision of children. Gu et al. (3) suggested that parental supervision might promote increasing MSE participation in children and adolescents. Despite the variation in the association between satisfaction with weight and MSE participation, the directions remained similar. Shi et al. (21) concluded that individuals who were satisfied with their weight might be more motivated to engage in MSE. This finding indicates that living with parents and satisfaction with weight could be viewed as factors related to MSE participation. However, more studies should be conducted to confirm the associations observed in the current study.

### 4.1 Study limitations

It is important to acknowledge certain limitations of this study. Due to its cross-sectional design, causality between variables cannot be established. While some demographic factors may have a unidirectional influence on MSE participation—meaning they impact MSE rather than MSE influencing them—other variables, such as sports club participation, may exhibit a bidirectional relationship. Specifically, students involved in sports clubs may be more likely to engage in MSE, while regular MSE participation might encourage students to seek structured sports environments. Future research employing longitudinal or experimental designs is necessary to explore the causal relationships between these correlates and MSE participation in children and adolescents and investigate the underlying mechanisms more deeply.

Additionally, data collection was based on self-reported questionnaires, making it susceptible to recall biases and the influence of social desirability, which may affect the interpretation of the results. Measurement challenges, such as incompletely capturing different MSE domains (e.g., upper or lower body, intensity) and excluding potentially confounding factors like parenting styles, could also impact the findings. Addressing these gaps in future research will enhance the understanding of MSE engagement and inform the development of targeted interventions.

### 4.2 Practical implications

The findings from this study have significant implications for public health interventions and policy. Recognizing the sex disparity in MSE participation, targeted strategies should be implemented to encourage greater engagement among girls through gender-sensitive approaches, such as designing MSE programs that align with their interests and motivations. Additionally, grade-appropriate interventions that leverage adolescents’ increasing autonomy should be developed to enhance adherence to MSE guidelines, ensuring that physical activity habits are sustained over time. Given the critical role of schools in promoting MSE, school-based interventions should integrate MSE into physical education curricula, providing structured opportunities for students to engage in strength-based activities (59, 60). Beyond the curriculum, schools should offer extracurricular programs that expose students to enjoyable and engaging sports, fostering a positive attitude toward lifelong physical activity. Furthermore, community-based initiatives must play a role in expanding access to safe and affordable exercise facilities, particularly for children from lower SES backgrounds, to mitigate disparities in MSE participation. Policymakers should ensure equitable distribution of resources to underserved schools and communities, addressing financial and environmental barriers that may hinder participation.

In addition to direct policy interventions, technological innovations should be explored to promote MSE engagement. Using fitness apps, gamification, and digital platforms could provide engaging and interactive ways to encourage participation, especially among youth accustomed to digital environments. Future research should further investigate the effectiveness of experimental interventions, including integrating digital tools, to determine their impact on MSE participation.

## 5 Conclusion

This study highlights the key factors influencing MSE participation among children and adolescents, emphasizing the need for inclusive public health strategies to bridge demographic and behavioral disparities. By identifying critical determinants such as sex, age, socioeconomic status, sports club involvement, and weight perception, the findings offer a foundation for designing targeted interventions that promote equitable access to MSE. Beyond individual health benefits, increasing MSE participation has broader societal implications, contributing to long-term physical well-being, reducing health disparities, and fostering lifelong active habits. Future research should adopt longitudinal approaches to deepen the understanding of these associations and inform more effective policy and educational initiatives.

## Data Availability

The original contributions presented in the study are included in the article/supplementary material, further inquiries can be directed to the corresponding authors.

## References

[1] Bull FC Al-Ansari SS Biddle S Borodulin K Buman MP Cardon G. World Health Organization 2020 guidelines on physical activity and sedentary behaviour. Br J Sports Med. (2020) 54:1451–62. doi: 10.1136/bjsports-2020-102955, PMID: 33239350 PMC7719906

[2] Xia R Yang L Liang C Lyu D Zang W Sun G. Research on aerobic fitness in children and adolescents: a bibliometric analysis based on the 100 most-cited articles. Front Med. (2024) 11:11. doi: 10.3389/fmed.2024.1409532, PMID: 39386747 PMC11461214

[3] Gu J Hong J-T Lin Y Yan J Chen S. Correlates of meeting the muscle-strengthening exercise guidelines in children and adolescent. Front Public Health. (2022) 10:854100. doi: 10.3389/fpubh.2022.854100 35712264 PMC9197157

[4] Xin F Zhu Z Chen S Chen H Hu X Ma X. Prevalence and correlates of meeting the muscle-strengthening exercise recommendations among Chinese children and adolescents: results from 2019 physical activity and fitness in China—the youth study. J Sport Health Sci. (2022) 11:358–66. doi: 10.1016/j.jshs.2021.09.010, PMID: 34606977 PMC9189699

[5] Chen S Yan J Zhao Y. A trend analysis of adherence to the muscle strengthening exercise guidelines in US adolescents. Int J Public Health. (2022) 67:1605022. doi: 10.3389/ijph.2022.1605022 36457827 PMC9705328

[6] Ren Z Zhang Y Drenowatz C Eather N Hong J Wang L. How many adults have sufficient muscle-strengthening exercise and the associated factors: a systematic review consisting of 2,629,508 participants. J. Exerc. Sci. Fitness. (2024) 22:359–68. doi: 10.1016/j.jesf.2024.06.002, PMID: 39040428 PMC11261455

[7] Piercy KL Troiano RP Ballard RM Carlson SA Fulton JE Galuska DA. The physical activity guidelines for Americans. JAMA. (2018) 320:2020–8. doi: 10.1001/jama.2018.14854, PMID: 30418471 PMC9582631

[8] World Health Organization. WHO guidelines on physical activity and sedentary behaviour World Health Organization (2020). 33369898

[9] Li H Zhang W Yan J. Physical activity and sedentary behavior among school-going adolescents in low- and middle-income countries: insights from the global school-based health survey. PeerJ. (2024) 12:e17097. doi: 10.7717/peerj.17097, PMID: 38680891 PMC11055511

[10] Liu S Yu Q Hossain M-M Doig S Bao R Zhao Y. Meeting 24-h movement guidelines is related to better academic achievement: findings from the YRBS 2019 cycle. Int J Ment Health Promot. (2022) 24:13–24. doi: 10.32604/IJMHP.2021.017660, PMID: 39055887

[11] Dankel SJ Loenneke JP Loprinzi PD. Combined associations of muscle-strengthening activities and accelerometer-assessed physical activity on multimorbidity: findings from NHANES. Am J Health Promot. (2016) 31:274–7. doi: 10.4278/ajhp.150520-QUAN-894, PMID: 26730562

[12] Kann L Mcmanus T Harris WA Shanklin SL Flint KH Queen B. Youth risk behavior surveillance — United States, 2017. MMWR Surveill Summ. (2018) 67:1–114. doi: 10.15585/mmwr.ss6708a1, PMID: 29902162 PMC6002027

[13] Bennie JA Faulkner G Smith JJ. The epidemiology of muscle-strengthening activity among adolescents from 28 European countries. Scand J Public Health. (2022) 50:295–302. doi: 10.1177/14034948211031392, PMID: 34304606

[14] Lin Y Yan J. Muscle-strengthening activities and sociodemographic correlates among adults: findings from samples in mainland China. Int J Environ Res Public Health. (2020) 17:2266. doi: 10.3390/ijerph17072266, PMID: 32230937 PMC7177312

[15] Golden SD McLeroy KR Green LW Earp JAL Lieberman LD. Upending the social ecological model to guide health promotion efforts toward policy and environmental. Change. (2015) 42:8S–14S. doi: 10.1177/1090198115575098 25829123

[16] Wilson OWA Smith M Duncan S Hinckson E Mizdrak A Richards J. Differences in physical activity participation among young adults in Aotearoa New Zealand. BMC Public Health. (2023) 23:150. doi: 10.1186/s12889-023-15063-6, PMID: 36690969 PMC9869605

[17] Hao Y Lyu D Zhang S Guo B Yan J. Sports participation and depressive symptoms in youth: demographic differences. Int J Ment Health Promot. (2024) 26:865–73. doi: 10.32604/ijmhp.2024.055231

[18] Murcia JAM Gimeno EC Lacárcel J Pérez L. Physical self-concept of Spanish schoolchildren: differences by gender, sport practice and levels of sport involvement. J. Educ. Hum. Dev. (2007) 1:1–17.

[19] Belhaidas MB Tahar T Yan J Atallah A O’Keeffe BT Eather N. Psychometric properties of the basketball throw test as a health-related field-based measure of muscular strength for use with adolescents in school settings. J Teach Phys Educ. (2025) doi: 10.1123/jtpe.2024-0207 [E-pub ahead of print]., PMID: 36626690

[20] Martini MCS Assumpção D Barros MBA Barros Filho AA Mattei J. Satisfaction with body weight among adolescents with excess weight: findings from a cross-sectional population-based study. Sao Paulo Med J. (2020) 138:377–84. doi: 10.1590/1516-3180.2020.0007.R1.10062020, PMID: 32965450 PMC9673857

[21] Shi J Gao M Xu X Zhang X Yan J. Associations of muscle-strengthening exercise with overweight, obesity, and depressive symptoms in adolescents: findings from 2019 youth risk behavior surveillance system. Front Psychol. (2022) 13:980076. doi: 10.3389/fpsyg.2022.980076 36160591 PMC9495934

[22] Effa CJ Dolgoy ND McNeely ML. Resistance exercise and art therapy on body image in breast cancer: a scoping review. Women’s Health Rep. (2020) 1:424–35. doi: 10.1089/whr.2020.0058, PMID: 33786507 PMC7784823

[23] Wang H Du H Guan Y Zhong J Li N Pan J. Association between frequency of muscle-strengthening exercise and depression symptoms among middle and high school students: cross-sectional survey study. JMIR Public Health Surveill. (2024) 10:e50996. doi: 10.2196/50996, PMID: 38630529 PMC11063876

[24] Parker K Salmon J Ridgers ND Sahlqvist S Uddin R Veitch J. Socioecological correlates associated with muscle-strengthening exercise at home during COVID-19 among adolescents: the our life at home study. J Sports Sci. (2022) 40:899–907. doi: 10.1080/02640414.2022.2028964, PMID: 35060843

[25] Toivo K Vähä-Ypyä H Kannus P Tokola K Alanko L Heinonen OJ. Physical activity measured by accelerometry among adolescents participating in sports clubs and non-participating peers. Eur J Sport Sci. (2023) 23:1426–34. doi: 10.1080/17461391.2022.2103740, PMID: 35861140

[26] Su H Lyu D Huang K Yan J. Association of physical activity, screen time and sleep with substance use in children and adolescents: a large sample cross-sectional study. Front Public Health. (2024) 12:12. doi: 10.3389/fpubh.2024.1432710, PMID: 39484350 PMC11524877

[27] Shi C Yan J Wang L Shen H. Exploring the self-reported physical fitness and self-rated health, mental health disorders, and body satisfaction among Chinese adolescents: a cross-sectional study. Front Psychol. (2022) 13:1003231. doi: 10.3389/fpsyg.2022.1003231, PMID: 36186394 PMC9521502

[28] Kokko S Martin L Geidne S Van Hoye A Lane A Meganck J. Does sports club participation contribute to physical activity among children and adolescents? A comparison across six European countries. Scand J Public Health. (2019) 47:851–8. doi: 10.1177/1403494818786110, PMID: 29999480

[29] Wiese CW Kuykendall L Tay L. Get active? A meta-analysis of leisure-time physical activity and subjective well-being. J Posit Psychol. (2018) 13:57–66. doi: 10.1080/17439760.2017.1374436, PMID: 40101104

[30] Liu T Li D Yang H Chi X Yan J. Associations of sport participation with subjective well-being: a study consisting of a sample of Chinese school-attending students. Front Pub Health (2023) 11:1199782. 10.3389/fpubh.2023.1199782 PMC10326896 37427269

[31] Bottolfs M Støa EM Reinboth MS Svendsen MV Schmidt SK Oellingrath IM. Resilience and lifestyle-related factors as predictors for health-related quality of life among early adolescents: a cross-sectional study. J Int Med Res. (2020) 48:300060520903656. doi: 10.1177/0300060520903656, PMID: 32070172 PMC7111039

[32] Hadland SE Austin SB Goodenow CS Calzo JP. Weight misperception and unhealthy weight control behaviors among sexual minorities in the general adolescent population. J Adolesc Health. (2014) 54:296–303. doi: 10.1016/j.jadohealth.2013.08.021 24182939 PMC3943999

[33] Cundiff JM Smith TW Uchino BN Berg CA. Subjective social status: construct validity and associations with psychosocial vulnerability and self-rated health. Int J Behav Med. (2013) 20:148–58. doi: 10.1007/s12529-011-9206-1, PMID: 22200973

[34] Smith JJ Diallo TMO Bennie JA Tomkinson GR Lubans DR. Factors associated with adherence to the muscle-strengthening activity guideline among adolescents. Psychol. Sport Exerc. (2020) 51:101747–8. doi: 10.1016/j.psychsport.2020.101747

[35] Allender S Cowburn G Foster C. Understanding participation in sport and physical activity among children and adults: a review of qualitative studies. Health Educ Res. (2006) 21:826–35. doi: 10.1093/her/cyl063, PMID: 16857780

[36] Cheng YJ Gregg EW De Rekeneire N Williams DE Imperatore G Caspersen CJ. Muscle-strengthening activity and its association with insulin sensitivity. Diabetes Care. (2007) 30:2264–70. doi: 10.2337/dc07-0372, PMID: 17586746

[37] Wang L Chen R. Psychological needs satisfaction, self-determined motivation, and physical activity of students in physical education: comparison across gender and school levels. Eur J Sport Sci. (2022) 22:1577–85. doi: 10.1080/17461391.2021.1978558, PMID: 34503401

[38] Casey MM Telford A Mooney A Harvey JT Eime RM Payne WR. Linking secondary school physical education with community sport and recreation for girls: a process evaluation. BMC Public Health. (2014) 14:1039. doi: 10.1186/1471-2458-14-1039, PMID: 25283157 PMC4196094

[39] Benitez TJ Artigas E Larsen B Joseph RP Pekmezi D Marquez B. Barriers and facilitators to muscle-strengthening activity among Latinas in the U.S.: results from formative research assessments. Int J Behav Med. (2024) 31:292–304. doi: 10.1007/s12529-023-10183-0, PMID: 37231222 PMC12965181

[40] Eather N Morgan PJ Lubans DR. Social support from teachers mediates physical activity behavior change in children participating in the Fit-4-fun intervention. Int J Behav Nutr Phys Act. (2013) 10:68. doi: 10.1186/1479-5868-10-68, PMID: 23714651 PMC3672071

[41] Zhang T Solmon MA Kosma M Carson RL Gu X. Need support, need satisfaction, intrinsic motivation, and physical activity participation among middle school students. J Teach Phys Educ. (2011) 30:51–68. doi: 10.1123/jtpe.30.1.51

[42] Chen S Liang K López-Gil JF Drenowatz C Tremblay MS. Association between meeting 24-h movement guidelines and academic performance in a sample of 67,281 Chinese children and adolescents. Eur J Sport Sci. (2024) 24:487–98. doi: 10.1002/ejsc.12034, PMID: 40145126

[43] Chen S-T Yan J. Prevalence and selected sociodemographic of movement behaviors in schoolchildren from low-and middle-income families in Nanjing, China: a cross-sectional questionnaire survey. Children. (2020) 7:13. doi: 10.3390/children7020013, PMID: 32069924 PMC7072474

[44] Skauge M Seippel Ø. Where do they all come from? Youth, fitness gyms, sport clubs and social inequality. Sport Soc. (2022) 25:1506–27. doi: 10.1080/17430437.2020.1840554

[45] Beets MW Cardinal BJ Alderman BL. Parental social support and the physical activity-related behaviors of youth: a review. Health Educ Behav. (2010) 37:621–44. doi: 10.1177/1090198110363884, PMID: 20729347

[46] Stalsberg R Pedersen AV. Effects of socioeconomic status on the physical activity in adolescents: a systematic review of the evidence. Scand J Med Sci Sports. (2010) 20:368–83. doi: 10.1111/j.1600-0838.2009.01047.x, PMID: 20136763

[47] Craggs C Corder K van Sluijs EMF Griffin SJ. Determinants of change in physical activity in children and adolescents: a systematic review. Am J Prev Med. (2011) 40:645–58. doi: 10.1016/j.amepre.2011.02.025, PMID: 21565658 PMC3100507

[48] Davison KK Lawson CT. Do attributes in the physical environment influence children's physical activity? A review of the literature. Int J Behav Nutr Phys Act. (2006) 3:19. doi: 10.1186/1479-5868-3-19, PMID: 16872543 PMC1557665

[49] Foster C Moore JB Singletary CR Skelton JA. Physical activity and family-based obesity treatment: A review of expert recommendations on physical activity in youth. Clin Obes. (2018) 8:68–79. 29224232 10.1111/cob.12230

[50] Bennie JA Lee D-C Khan A Wiesner GH Bauman AE Stamatakis E. Muscle-strengthening exercise among 397,423 U.S. adults: prevalence, correlates, and associations with health conditions. Am J Prev Med. (2018) 55:864–74. doi: 10.1016/j.amepre.2018.07.022, PMID: 30458949

[51] Biddle SJH Asare M. Physical activity and mental health in children and adolescents: a review of reviews. Br J Sports Med. (2011) 45:886–95. doi: 10.1136/bjsports-2011-090185, PMID: 21807669

[52] Janssen I LeBlanc AG. Systematic review of the health benefits of physical activity and fitness in school-aged children and youth. Int J Behav Nutr Phys Act. (2010) 7:40. doi: 10.1186/1479-5868-7-40, PMID: 20459784 PMC2885312

[53] Hertwig R Davis JN Sulloway FJ. Parental investment: how an equity motive can produce inequality. Psychol Bull. (2002) 128:728–45. doi: 10.1037/0033-2909.128.5.728, PMID: 12206192

[54] Sawka KJ McCormack GR Nettel-Aguirre A Hawe P Doyle-Baker PK. Friendship networks and physical activity and sedentary behavior among youth: a systematized review. Int J Behav Nutr Phys Act. (2013) 10:1–9. doi: 10.1186/1479-5868-10-130, PMID: 24289113 PMC4220781

[55] Park SH Cormier E. Influence of siblings on child health behaviors and obesity: a systematic review. J Child Fam Stud. (2018) 27:2069–81. doi: 10.1007/s10826-018-1049-9, PMID: 40144921

[56] Fitzgerald A Fitzgerald N Aherne C. Do peers matter? A review of peer and/or friends’ influence on physical activity among American adolescents. J Adolesc. (2012) 35:941–58. doi: 10.1016/j.adolescence.2012.01.002, PMID: 22285398

[57] Qurban H Wang J Siddique H Morris T Qiao Z. The mediating role of parental support: the relation between sports participation, self-esteem, and motivation for sports among chinese students. Curr Psychol. (2019) 38:308–19. doi: 10.1007/s12144-018-0016-3

[58] Ramer JD DuBois DL Duncan RJ Bustamante AS Vandell DL Marquez DX. Childhood predictors of high school sport participation and effects of participation on young adult activity and mental health. Ann Med. (2025) 57:2447905. doi: 10.1080/07853890.2024.2447905, PMID: 39746664 PMC11703372

[59] Yan J Morgan PJ Smith JJ Chen S Leahy AA Eather N. Pilot randomized controlled trial of a game-based intervention for teaching basketball in Chinese primary school physical education. J Sports Sci. (2024) 42:25–37. doi: 10.1080/02640414.2024.2319457, PMID: 38381852

[60] Yan J Jones B Smith JJ Morgan P Eather N. A systematic review investigating the effects of implementing game-based approaches in school-based physical education among primary school children. J Teach Phys Educ. (2023) 42:573–86. doi: 10.1123/jtpe.2021-0279, PMID: 36626690

